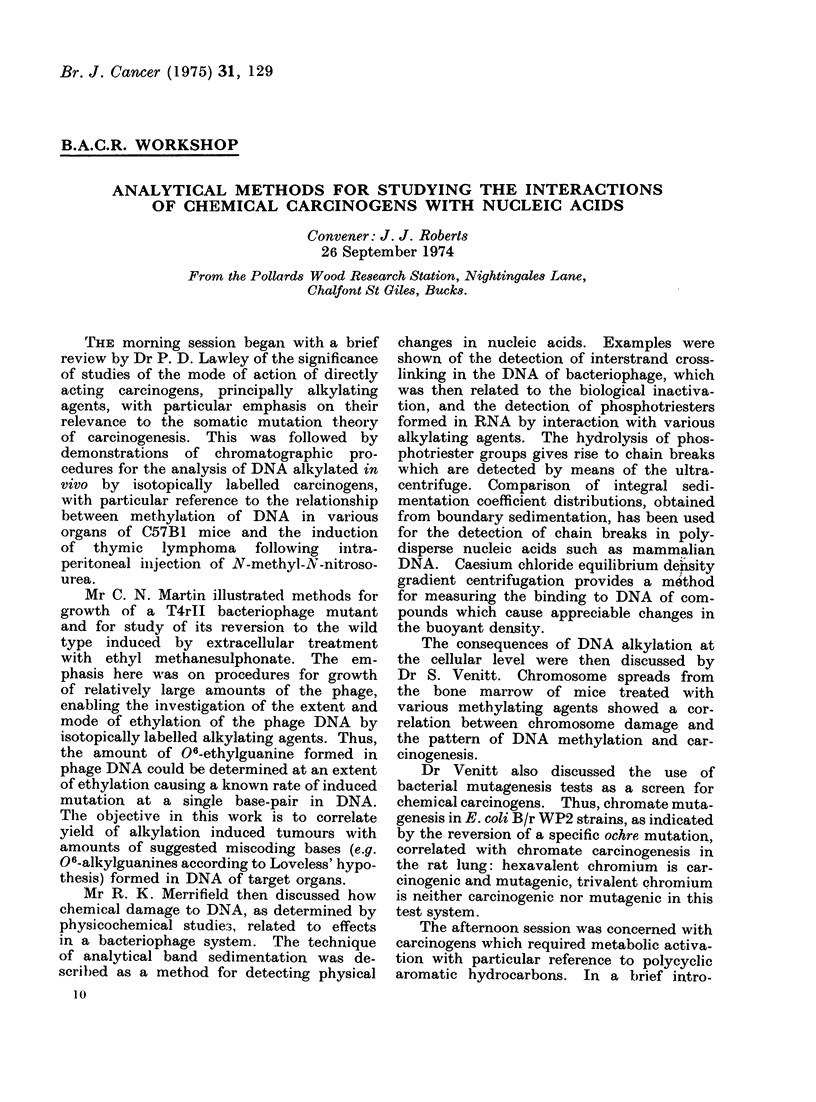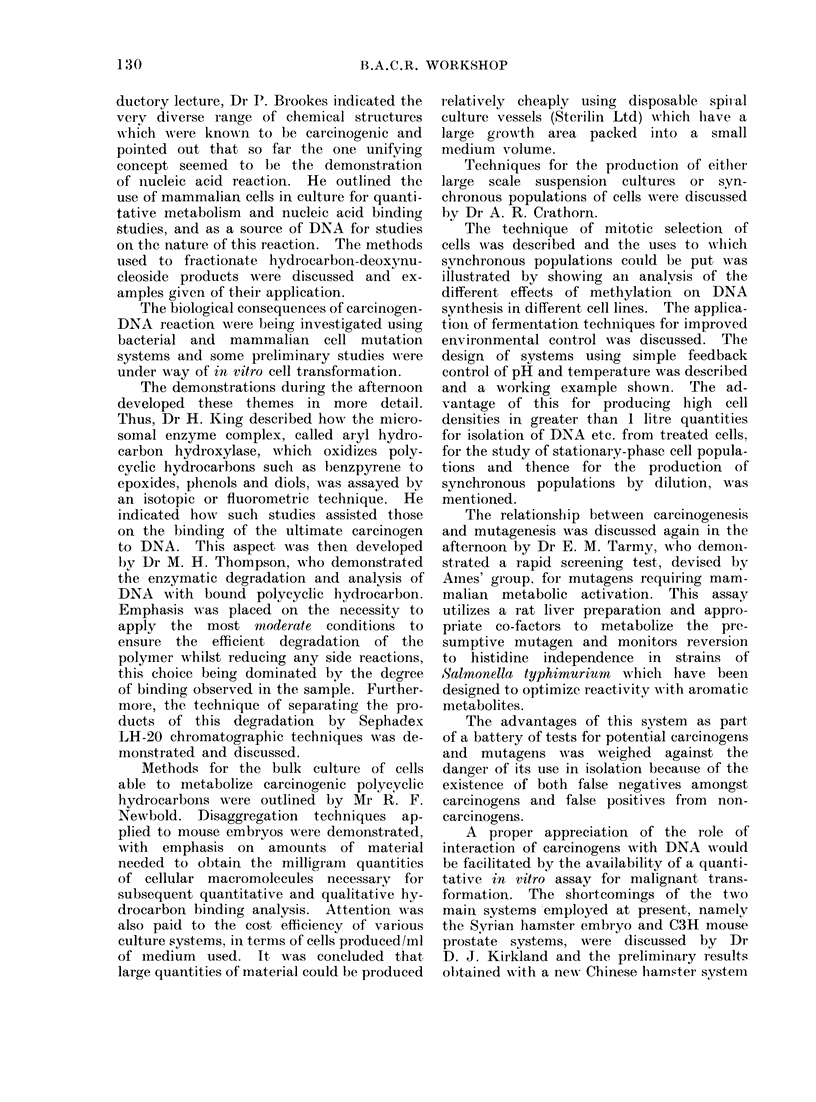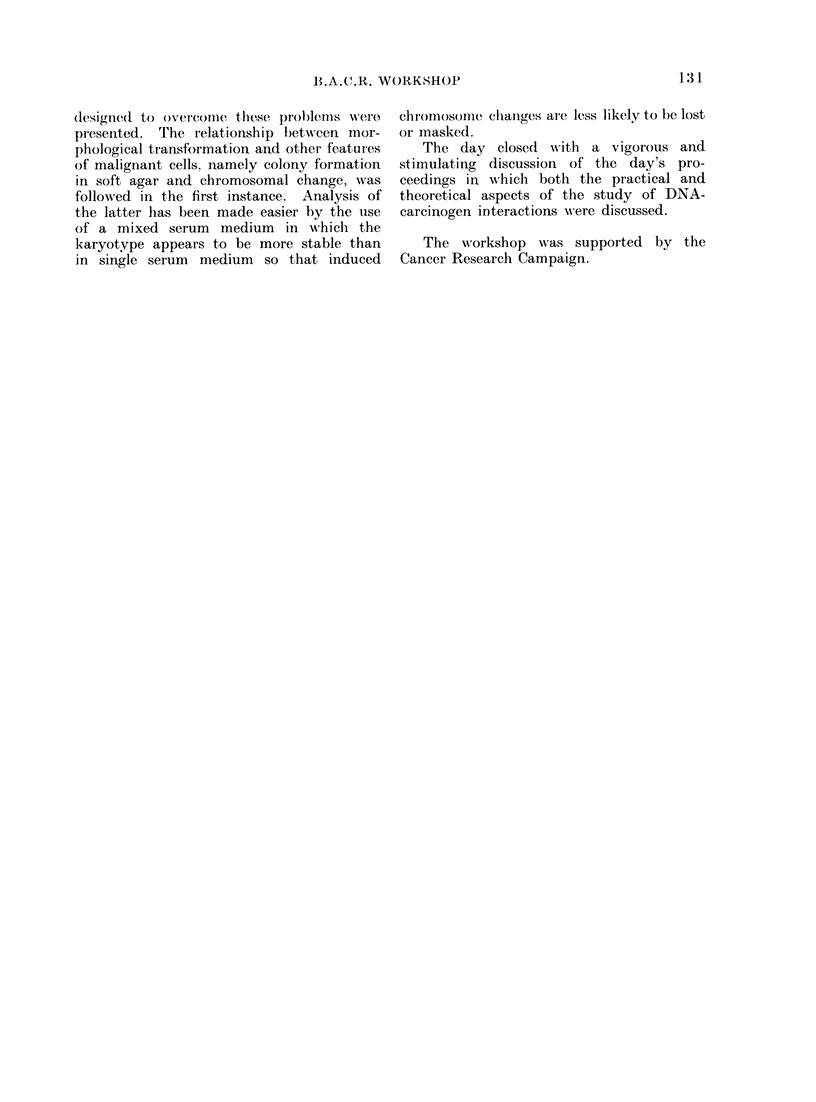# Analytical Methods for Studying the Interactions of Chemical Carcinogens with Nucleic Acids

**Published:** 1975-01

**Authors:** 


*Br. J. Cancer* (1975) **31**, 129

### B.A.C.R. WORKSHOP

### ANALYTICAL METHODS FOR STUDYING THE INTERACTIONS OF CHEMICAL CARCINOGENS WITH NUCLEIC ACIDS

*Convener: J. J. Roberts*
26 September 1974

*From the Pollards Wood Research Station, Nightingales Lane, Chalfont St Giles, Bucks.*

THE morning session began with a brief review by Dr P. D. Lawley of the significance of studies of the mode of action of directly acting carcinogens, principally alkylating agents, with particular emphasis on their relevance to the somatic mutation theory of carcinogenesis. This was followed by demonstrations of chromatographic procedures for the analysis of DNA alkylated *in vivo* by isotopically labelled carcinogens, with particular reference to the relationship between methylation of DNA in various organs of C57Bl mice and the induction of thymic lymphoma following intraperitoneal injection of *N*-methyl-*N*-nitrosourea.

Mr C. N. Martin illustrated methods for growth of a T4rII bacteriophage mutant and for study of its reversion to the wild type induced by extracellular treatment with ethyl methanesulphonate. The emphasis here was on procedures for growth of relatively large amounts of the phage, enabling the investigation of the extent and mode of ethylation of the phage DNA by isotopically labelled alkylating agents. Thus, the amount of $O^6$-ethylguanine formed in phage DNA could be determined at an extent of ethylation causing a known rate of induced mutation at a single base-pair in DNA. The objective in this work is to correlate yield of alkylation induced tumours with amounts of suggested miscoding bases (*e.g.* $O^6$-alkylguanines according to Loveless' hypothesis) formed in DNA of target organs.

Mr R. K. Merrifield then discussed how chemical damage to DNA, as determined by physicochemical studies, related to effects in a bacteriophage system. The technique of analytical band sedimentation was described as a method for detecting physical changes in nucleic acids. Examples were shown of the detection of interstrand cross-linking in the DNA of bacteriophage, which was then related to the biological inactivation, and the detection of phosphotriesters formed in RNA by interaction with various alkylating agents. The hydrolysis of phosphotriester groups gives rise to chain breaks which are detected by means of the ultracentrifuge. Comparison of integral sedimentation coefficient distributions, obtained from boundary sedimentation, has been used for the detection of chain breaks in polydisperse nucleic acids such as mammalian DNA. Caesium chloride equilibrium density gradient centrifugation provides a method for measuring the binding to DNA of compounds which cause appreciable changes in the buoyant density.

The consequences of DNA alkylation at the cellular level were then discussed by Dr S. Venitt. Chromosome spreads from the bone marrow of mice treated with various methylating agents showed a correlation between chromosome damage and the pattern of DNA methylation and carcinogenesis.

Dr Venitt also discussed the use of bacterial mutagenesis tests as a screen for chemical carcinogens. Thus, chromate mutagenesis in *E. coli* B/r WP2 strains, as indicated by the reversion of a specific *ochre* mutation, correlated with chromate carcinogenesis in the rat lung: hexavalent chromium is carcinogenic and mutagenic, trivalent chromium is neither carcinogenic nor mutagenic in this test system.

The afternoon session was concerned with carcinogens which required metabolic activation with particular reference to polycyclic aromatic hydrocarbons. In a brief introductory lecture, Dr P. Brookes indicated the very diverse range of chemical structures which were known to be carcinogenic and pointed out that so far the one unifying concept seemed to be the demonstration of nucleic acid reaction. He outlined the use of mammalian cells in culture for quantitative metabolism and nucleic acid binding studies, and as a source of DNA for studies on the nature of this reaction. The methods used to fractionate hydrocarbon-deoxynucleoside products were discussed and examples given of their application.

The biological consequences of carcinogen-DNA reaction were being investigated using bacterial and mammalian cell mutation systems and some preliminary studies were under way of *in vitro* cell transformation.

The demonstrations during the afternoon developed these themes in more detail. Thus, Dr H. King described how the microsomal enzyme complex, called aryl hydrocarbon hydroxylase, which oxidizes polycyclic hydrocarbons such as benzpyrene to epoxides, phenols and diols, was assayed by an isotopic or fluorometric technique. He indicated how such studies assisted those on the binding of the ultimate carcinogen to DNA. This aspect was then developed by Dr M. H. Thompson, who demonstrated the enzymatic degradation and analysis of DNA with bound polycyclic hydrocarbon. Emphasis was placed on the necessity to apply the most *moderate* conditions to ensure the efficient degradation of the polymer whilst reducing any side reactions, this choice being dominated by the degree of binding observed in the sample. Furthermore, the technique of separating the products of this degradation by Sephadex LH-20 chromatographic techniques was demonstrated and discussed.

Methods for the bulk culture of cells able to metabolize carcinogenic polycyclic hydrocarbons were outlined by Mr R. F. Newbold. Disaggregation techniques applied to mouse embryos were demonstrated, with emphasis on amounts of material needed to obtain the milligram quantities of cellular macromolecules necessary for subsequent quantitative and qualitative hydrocarbon binding analysis. Attention was also paid to the cost efficiency of various culture systems, in terms of cells produced/ml of medium used. It was concluded that large quantities of material could be produced relatively cheaply using disposable spiral culture vessels (Sterilin Ltd) which have a large growth area packed into a small medium volume.

Techniques for the production of either large scale suspension cultures or synchronous populations of cells were discussed by Dr A. R. Crathorn.

The technique of mitotic selection of cells was described and the uses to which synchronous populations could be put was illustrated by showing an analysis of the different effects of methylation on DNA synthesis in different cell lines. The application of fermentation techniques for improved environmental control was discussed. The design of systems using simple feedback control of pH and temperature was described and a working example shown. The advantage of this for producing high cell densities in greater than 1 litre quantities for isolation of DNA etc. from treated cells, for the study of stationary-phase cell populations and thence for the production of synchronous populations by dilution, was mentioned.

The relationship between carcinogenesis and mutagenesis was discussed again in the afternoon by Dr E. M. Tarmy, who demonstrated a rapid screening test, devised by Ames' group, for mutagens requiring mammalian metabolic activation. This assay utilizes a rat liver preparation and appropriate co-factors to metabolize the presumptive mutagen and monitors reversion to histidine independence in strains of *Salmonella typhimurium* which have been designed to optimize reactivity with aromatic metabolites.

The advantages of this system as part of a battery of tests for potential carcinogens and mutagens was weighed against the danger of its use in isolation because of the existence of both false negatives amongst carcinogens and false positives from non-carcinogens.

A proper appreciation of the role of interaction of carcinogens with DNA would be facilitated by the availability of a quantitative *in vitro* assay for malignant transformation. The shortcomings of the two main systems employed at present, namely the Syrian hamster embryo and C3H mouse prostate systems, were discussed by Dr D. J. Kirkland and the preliminary results obtained with a new Chinese hamster system designed to overcome these problems were presented. The relationship between morphological transformation and other features of malignant cells, namely colony formation in soft agar and chromosomal change, was followed in the first instance. Analysis of the latter has been made easier by the use of a mixed serum medium in which the karyotype appears to be more stable than in single serum medium so that induced chromosome changes are less likely to be lost or masked.

The day closed with a vigorous and stimulating discussion of the day's proceedings in which both the practical and theoretical aspects of the study of DNA-carcinogen interactions were discussed.

The workshop was supported by the Cancer Research Campaign.